# Patient-reported burden of hereditary transthyretin amyloidosis on functioning and well-being

**DOI:** 10.1186/s41687-020-00273-y

**Published:** 2021-01-07

**Authors:** Andrew Lovley, Kimberly Raymond, Spencer D. Guthrie, Michael Pollock, Vaishali Sanchorawala, Michelle K. White

**Affiliations:** 1 QualityMetric Incorporated, LLC, 1301 Atwood Avenue, Suite 216E, Johnston, RI 02919 USA; 2 Aurora Bio, San Francisco, CA USA; 3 Akcea Therapeutics, Boston, MA USA; 4 Amyloidosis Center, Boston University School of Medicine and Boston Medical Center, Boston, MA USA

**Keywords:** Amyloidosis, Burden of disease, Quality of life, Patient interviews, Qualitative, Rare disease

## Abstract

**Background:**

Hereditary transthyretin (hATTR) amyloidosis is a rare, systemic, progressive, and life-threatening disease in which transthyretin proteins misfold and aggregate as insoluble amyloid deposits, disrupting nervous, cardiac, gastrointestinal, and other organ tissues. There are limited available data about the experience of patients living with hATTR amyloidosis. This study used a qualitative, non-interventional design to explore the humanistic burden of hATTR amyloidosis from the patient’s perspective.

**Results:**

Fourteen adults with hATTR amyloidosis, recruited from a patient advocacy group or an academic clinical center, participated in individual semi-structured interviews either in person or by telephone. Patients were asked to describe their experiences living with the condition, including symptoms and disease-related impacts on functioning and well-being, work, and activities of daily living (ADLs). Interviews were transcribed verbatim and analyzed for key concepts using a grounded theory approach.

Patients described many symptoms of hATTR amyloidosis, particularly those associated with peripheral neuropathy such as pain, numbness, weakness, and paresthesia. Symptoms of autonomic neuropathy, such as gastrointestinal dysfunction, and symptoms related to cardiac dysfunction were also common. Worsening symptoms, especially those impacting patients’ ability to walk or use their hands, often led to a loss of autonomy and an inability to work or perform ADLs. Disease-related disability also interfered with patients’ participation in social activities, and contributed to feelings of fear, frustration, or sadness.

**Conclusions:**

The impacts of hATTR amyloidosis were profound for the patients interviewed for this study. They described a sense of loss as their condition progressed and impacted them physically, emotionally, and socially. Patients’ reports of symptoms and impacts of hATTR amyloidosis illustrate the complex and varied manifestations of this disease. The progression of symptoms and increasing impacts of hATTR amyloidosis also highlight the need for an earlier diagnosis and effective clinical intervention to preserve patients’ functioning and well-being.

## Background

Hereditary transthyretin (hATTR) amyloidosis is a rare, systemic, progressive, and life-threatening disease in which transthyretin (TTR) proteins misfold and aggregate as insoluble amyloid deposits, disrupting nervous, cardiac, and other organ tissues throughout the body [1, 2]. Over 140 mutations of the TTR gene that cause TTR proteins to misfold have been identified, each of which are associated with different phenotypic manifestations [3]. The prevalence of hATTR amyloidosis has been estimated to be approximately 50,000 patients worldwide [4]. Signs and symptoms of hATTR amyloidosis typically present between 30 and 70 years of age, and the survival rate is 3–15 years after diagnosis, though onset and survival can vary widely by genetic mutation and clinical phenotype [4, 5].

As the disease progresses, hATTR amyloidosis manifests primarily as symptoms of polyneuropathy (PN) and/or cardiomyopathy (CM), frequently leading to sensorimotor impairment, gastrointestinal or other autonomic dysfunction, or heart failure, among many other symptoms [5, 6]. The systemic nature and broad impact of the disease is associated with substantial deficits in health-related quality of life (HRQoL) [7–9]. Patients with hATTR amyloidosis accompanied by PN eventually experience disability with ambulation and using their arms and hands (e.g., difficulty lifting objects, loss of fine motor skills). Patients who have hATTR amyloidosis with CM typically experience symptoms such as fatigue, arrhythmia, and shortness of breath [10].

Symptoms experienced by patients with hATTR amyloidosis can interfere with their ability to work and complete typical household chores. Symptomatic patients frequently report being unable to work [11, 12] and those who do continue to work commonly experience challenges with absenteeism and presenteeism [7]. In patients’ domestic lives, hATTR amyloidosis is associated with deficits on measures of role functioning and the ability to carry out activities of daily living (ADLs) [7, 8].

Much is still unknown about the experience of patients with hATTR amyloidosis. To date, the burden of hATTR amyloidosis on patients’ functioning and well-being has been assessed exclusively with the use of standardized survey instruments [7–9]. To develop a more comprehensive understanding of hATTR amyloidosis, it is important that the voice of the patient is taken into account. Qualitative interview methods allow for a deep exploration of how patients are affected by symptoms and impacts of the disease in ways that go beyond the limitations of multiple-choice responses used in quantitative survey studies. To that end, this qualitative study aimed to describe the burden of hATTR amyloidosis on functioning and well-being from the patient’s perspective.

## Methods

### Study design

This was a qualitative, non-interventional study designed to explore the symptoms of hATTR amyloidosis and their impact on patients’ functioning and well-being. Individual interviews, each lasting between 60 and 70 min, were conducted with patients by phone (*n* = 12) or in person (*n* = 2). All study materials, including the study protocol, interview guide, informed consent form (ICF), and recruitment flyers, were approved by the New England Independent Review Board (NEIRB) prior to the start of the study.

### Patient population and recruitment

Patients were eligible to participate in the study if they reported that they had received a diagnosis of hATTR amyloidosis and could communicate fluently in English. Patients were excluded from participation if they had a diagnosis of wild-type ATTR amyloidosis (i.e., a non-genetic form of ATTR amyloidosis), or if they were unable to sit for 70 min to complete the interview. Recruitment of a convenience sample was facilitated by the Amyloidosis Center at Boston University School of Medicine and Boston Medical Center (BMC), a recognized center of excellence for the clinical diagnosis and treatment of amyloidosis, and the Amyloidosis Support Groups (ASG), a patient advocacy group whose aim is to offer support to patients, families, and caregivers affected by amyloidosis. Recruitment flyers were distributed to patients with hATTR amyloidosis. Patients who responded to the flyers were screened to verify eligibility, and those who were eligible to participate were scheduled for an interview. A target sample size of 15 was set in line with guidelines regarding the adequate number of interviews needed to reach saturation of a concept in qualitative patient interviews [13, 14]. Due to a slow in recruitment, the decision was made after 14 interviews to proceed with analysis.

### Study procedures

Individual interviews were conducted by one of two qualitative scientists between April and June of 2018. All patients provided written informed consent to participate in recorded interviews. During the interviews, patients were asked open-ended questions designed to elicit spontaneously reported descriptions of their experience of hATTR amyloidosis, including symptoms and impacts on aspects of their lives, such as physical functioning, emotional well-being, social functioning, ADLs, and work. The inclusion of open-ended questions within the interview guide helped focus the interview, while the inclusion of additional probes allowed researchers to collect more detailed information and expand on key components of the experiences under study [13]. Questions about frequency, severity, duration, quality, or other attributes of symptoms and experiences were purposely vague in order to not influence responses.

### Data coding and analysis

Audio recordings of the interviews were transcribed verbatim. Five transcripts were randomly selected and checked against the audio recordings to ensure transcription accuracy; no major omissions or problems were identified. All transcripts were content coded using NVivo qualitative research software (QSR International Pty Ltd. Version 11, 2015).

The analytic approach for this study was content thematic analysis [15]. This approach is in accordance with the principles of grounded theory, an inductive methodology whereby concepts emerge from patients rather than researchers imposing an a priori hypothesis [16]. Data were reviewed for emerging themes to identify patterns in responses concerning relevant and important aspects of patients’ experiences. In-depth discussions took place among research team members at regular intervals to resolve differences and agree upon the identification of any newly emerging concepts. No discrepancies were identified and consensus among research team members was easily achieved. An analysis of saturation (i.e., the point at which no new relevant information emerges) was conducted to confirm that enough interviews were completed to fully understand concepts important to patients [16].

## Results

### Saturation analysis

A total of 55 concepts emerged from the interviews across the five major themes selected for saturation evaluation (Table 1). Overall, 41 (75%) of the identified concepts emerged in the first four interviews, with an additional 10 (18%) concepts emerging in the next set of four interviews. All concepts relating to the impact of hATTR amyloidosis on patients’ HRQoL were elicited in the first two sets of interviews. Only four new concepts (7%) emerged in the last two sets of interviews, consisting primarily of symptoms that patients uncommonly attributed to hATTR amyloidosis, suggesting that 14 interviews were sufficient to reach saturation and that additional interviews would not have resulted in additional themes.

**Table 1:** Saturation analysis: number of new concepts identified per interview set across each major theme

Major Theme	Interviews 1–4	Interviews 5–8	Interviews 9–12	Interviews 13–14
Symptoms	21	8	3	1
Impact on Physical Functioning	6	1	0	0
Impact on Emotional Well-being	4	0	0	0
Impact on Social Functioning	4	0	0	0
Impact on Work and Activities of Daily Living	6	1	0	0
**Total**	**41**	**10**	**3**	**1**

### Demographic and disease characteristics

Patients’ demographic and disease characteristics (*n* = 14) are shown in Table 2. Patients ranged from 20 to 76 years of age (mean = 61.5; SD = 14.5) and 57% were male. Most of the patients interviewed were Caucasian/white (*n* = 13; 93%) and held at least a four-year college degree (*n* = 11; 79%). All patients reported having been diagnosed with hATTR amyloidosis with symptoms of either PN alone (*n* = 6; 43%) or both PN and CM (*n* = 8; 57%); no patients reported symptoms of CM alone. Roughly half of patients (*n* = 8; 57%) reported being able to walk unassisted, while 43% (*n* = 6) reported being able to walk only with assistance or unable to walk at all.

**Table 2:** Demographic and disease characteristics of study sample

**Patients with hATTR Amyloidosis (*N*** **= 14)**	
	**Mean (SD) [Range]**
**Age (years)**	61.5 (14.5) [20–76]
**Age at First hATTR Amyloidosis Symptom (years)**	50.36 (15.13) [14–73]
**Age at Diagnosis (years)**	56.29 (16.55) [17–75]
**Time Since Diagnosis (years)**	5.21 (5.63) [1–21]
	***N*** **(%)**
**Sex**	
Male	8 (57%)
Female	6 (43%)
**Race**	
Caucasian/white	13 (93%)
Asian	1 (7%)
**Education**	
High school	1 (7%)
Some college but no degree	1 (7%)
Associate’s degree or technical certificate	1 (7%)
Bachelor’s degree	7 (50%)
Graduate degree	4 (29%)
**Marital Status**	
Married	10 (71%)
Divorced	3 (21%)
Never married	1 (7%)
**Region of Residence**	
Northeast US	5 (36%)
South US	5 (36%)
West US	2 (14%)
Midwest US	1 (7%)
Canada	1 (7%)
**hATTR Amyloidosis Type**	
hATTR amyloidosis with symptoms of both PN and CM	8 (57%)
hATTR amyloidosis with symptoms of PN only	6 (43%)
**Genetic Variant of hATTR Amyloidosis**	
V30M	5 (36%)
T60A	3 (21%)
Other^a^	4 (29%)
Unsure	2 (14%)
**Age at Symptom Onset**	
Early onset (< 50 years of age)	7 (50%)
Late onset (≥50 years of age)	7 (50%)
**Stage of Ambulatory Disability**	
Stage 1: Able to walk unassisted	8 (57%)
Stage 2: Requires assistance with walking (e.g., walker, cane)	3 (21%)
Stage 3: Unable to walk (e.g., wheelchair, scooter)	3 (21%)
**Impacted Organs/Systems**^**b**^	
Nervous	14 (100%)
Cardiac	8 (57%)
Gastrointestinal	8 (57%)
Ocular	4 (29%)

*Abbreviations*: *CM* Cardiomyopathy, *PN* Polyneuropathy, *SD* Standard deviation, *US* United States

^a^Other genetic variants reported by patients: A97S (*n* = 1), Y114H (*n* = 1), F64L (*n* = 1), E54G (*n* = 1)

^b^Because patients could report multiple organ/system involvement, frequency sums to > 100%

### Signs and symptoms

A total of 33 different signs and symptoms attributed to hATTR amyloidosis were reported by patients interviewed for this study (Table 3). All patients described symptoms of peripheral neuropathy such as pain (*n* = 12; 86%) or numbness (*n* = 12; 86%), either in general or localized to certain areas of their bodies such as the upper or lower limbs. Many patients reported fatigue and lethargy (*n* = 12; 86%) or weakness (*n* = 10; 71%). Other common symptoms included gastrointestinal dysfunction (*n* = 8; 57%) with constipation and diarrhea being the main complaints; cognitive dysfunction (*n* = 8; 57%), manifesting as trouble speaking, difficulty focusing, memory loss, preoccupation, or reduced processing speed; paresthesia (*n* = 8; 57%), including tingling in the arms, hands, legs, or feet; and cardiac dysfunction (*n* = 8; 57%), such as evidence of CM, palpitations, either high or low blood pressure, and rapid heartbeat. Patients who were probed about which symptoms they considered most bothersome (*n* = 10) cited pain (*n* = 4; 40%) and loss of balance (*n* = 4; 40%), as well as the loss of muscle control (*n* = 3; 30%), numbness (*n* = 2; 20%) and paresthesia (*n* = 2; 20%).

**Table 3:** Signs and Symptoms Associated with hATTR Amyloidosis

Symptom^**a**^	Patient^**b**^														
	01	02	03	04	05	06	07	08	09	10	11	12	13	14	Total
Pain	╳		╳	╳	╳		╳	╳	╳	╳	╳	╳	╳	╳	12
Numbness	╳		╳	╳		╳	╳	╳	╳	╳	╳	╳	╳	╳	12
Fatigue, Lethargy	╳	╳	╳		╳	╳	╳	╳	╳	╳	╳	╳		╳	12
Weakness	╳		╳	╳		╳		╳	╳	╳		╳	╳	╳	10
Gastrointestinal dysfunction	╳		╳	╳	╳			╳		╳	╳			╳	8
Cognitive dysfunction		╳	╳		╳	╳		╳		╳		╳		╳	8
Paresthesia	╳	╳	╳			╳		╳	╳			╳	╳		8
Cardiac dysfunction			╳	╳			╳	╳		╳	╳	╳		╳	8
Loss of motor function	╳		╳	╳		╳	╳		╳					╳	7
Loss of balance	╳					╳			╳	╳	╳			╳	6
Carpal tunnel syndrome				╳		╳		╳		╳	╳	╳			6
Dizziness				╳	╳	╳		╳						╳	5
Genitourinary dysfunction	╳		╳			╳								╳	4
Ocular issues	╳		╳			╳						╳			4
Difficulty breathing			╳							╳		╳		╳	4
Swelling									╳				╳	╳	3
Weight loss or difficulty gaining				╳	╳				╳						3
Hot sensations	╳		╳												2
Difficulty swallowing, Choking						╳								╳	2
**Total**	11	3	13	9	6	12	5	10	9	10	7	10	5	14	

^a^Fourteen symptoms were mentioned by only one patient each: altered taste, back spasms, body temperature dysregulation, elevated adrenaline, heat intolerance, inability to get pregnant, insomnia, migraine, muscle tightness, nausea, perspiration, sensitivity to weather, blood pooling, and weakened voice

^b^Genetic variants reported by patients: V30M (patient #s 01, 02, 03, 05, and 07), T60A (#s 11, 12, 14), A97S (#04), Y114H (#08), F64L (#09), E54G (#10), and unsure (#s 06 and 13)

All patients (*n* = 14; 100%) described the effects of hATTR amyloidosis as worsening over time, with some patients experiencing significant decline in a matter of months. Many patients (*n* = 10; 71%) expressed some uncertainty as to whether specific signs or symptoms were due to hATTR amyloidosis, particularly symptoms that were experienced prior to receiving their diagnosis. Common symptoms of hATTR amyloidosis such as pain, numbness, and fatigue were often attributed by patients, at least initially, to aging, treatment side effects, work or sports-related injury, or other known co-morbid conditions. Sometimes patients would seem baffled when trying to identify the cause of their symptoms.

I felt so horrible, and I had no idea [why], when I had always been so healthy before. Your mind is just like, ‘What the heck is going on?’ [#02, Female, Age 48, V30M]

### Impacts of hATTR amyloidosis on functioning and well-being

Patients reported a number of physical, emotional, and social impacts resulting from having hATTR amyloidosis. Worsening symptoms often led to a loss of autonomy and an inability to work or perform ADLs, which in turn resulted in complex role changes in their relationships with spouses, partners, and children. Patients described a loss of their ability to participate in activities that previously brought joy and meaning into their lives, such as fishing, going out for dinner with their spouse, playing with their grandchildren. The impacts of hATTR amyloidosis on functioning and well-being reported by patients are shown in Table 4.

**Table 4:** Impacts of hATTR Amyloidosis on patients’ functioning and well-being

Impact^a^	***N*** (%)
**Physical Functioning**	
Difficulty gripping/holding	11 (79%)
Difficulty walking/standing	11 (79%)
Difficulty bending/kneeling	5 (36%)
Difficulty climbing stairs/ladders	5 (36%)
Difficulty lifting heavy items	4 (29%)
Difficulty exercising	4 (29%)
Idleness	4 (29%)
**Emotional Well-being**	
Fear/anxiety	10 (71%)
Frustration/disappointment	7 (50%)
Sadness/depression	6 (43%)
Anguish/despair	4 (29%)
**Social Functioning**	
Unable to participate in social activities	11 (79%)
Misunderstood by others	4 (29%)
Difficulty communicating	2 (12%)
Self-isolation	2 (12%)
**Work and Activities of Daily Living**	
Early retirement	8 (57%)
Absenteeism	8 (57%)
Presenteeism	7 (50%)
Difficulty dressing, bathing, using bathroom	9 (64%)
Difficulty performing household chores	8 (57%)
Difficulty driving	8 (57%)
Difficulty eating	3 (21%)

^a^Because patients could report multiple impacts, frequency sums to > 100%

### Impacts of hATTR amyloidosis on physical functioning

Patients reported a variety of ways in which hATTR amyloidosis affected their physical functioning. In particular, patients described difficulties gripping and holding with their hands (*n* = 11; 79%) and difficulties walking or standing (*n* = 11; 79%), as well as difficulties with bending and kneeling (*n* = 5; 36%), climbing stairs or ladders (*n* = 5; 36%), and lifting heavy items (*n* = 4; 29%). The physical impacts of their condition made it difficult for some to exercise (*n* = 4; 29%) and in some cases forced them into idleness (*n* = 4; 29%).

Patients who experienced problems gripping with their hands reported frequently dropping things and having difficulties opening jars and bottles, holding a pen, holding a phone, using a computer, or gripping a steering wheel to drive their vehicle. Decreased mobility, such as the inability to walk or the loss of balance, impeded patients’ ability to move about their home and use stairs or ladders, run errands, lift and carry objects, or use the pedals in their vehicles. Some patients adopted the use of canes, walkers, or wheelchairs to help overcome reduced mobility, but nevertheless felt limited in what they could do as a result.

The thing that bother[s] me the most I think is my mobility. […] I can’t do the things I want to do like moving around, going places—I need assistance [from] people […] I need assistance from them to push me around [in my wheelchair]. That’s my biggest setback. And sometimes I look at people [who are] able to walk and say [to myself] ‘Why me?’ [#04, Male, Age 65, A97S]

Patients’ physical deficits, and inability to continue engaging in activities they had done previously, challenged how they perceived themselves and how they were perceived by others. Requiring extra assistance from others to complete certain physical tasks forced unexpected, and at times uncomfortable, role changes between patients and their spouses or family members.

If I drop something, it takes a long time for me to bend over slowly to pick it up, and then I ask my daughter ‘Oh, could you pick up that for me, […] can you do this for me?’ [and she replies] “Well, why don't you do it yourself? You look healthy.” […] I keep reminding [her], ‘Honey, I cannot do things’ and she thinks that I’m making her do all this work, picking things up and lifting things. I can’t open jars. My fingers [and] thumbs don't work. I've got butterfingers. I drop things. [#05, Female, Age 52, V30M]

### Impacts of hATTR amyloidosis on emotional well-being

Living with a progressive and degenerative disease caused emotional strain for many patients. Patients spontaneously described a variety of emotional responses relating to their multifaceted experience of the disease. Half of the patients (*n* = 7; 50%) described feelings of frustration and disappointment related to their hATTR amyloidosis, particularly with the progression of their symptoms, further loss of functioning, treatment not performing as they had hoped, or with having to stop working.

I was disgusted with [the disease] because I couldn’t drive anymore, and I had trouble walking, but I felt good about finding out what I had. … I mean, I get depressed because I can’t do anything like I did before. My life [has] changed completely around. [#09, Male, Age 76, F64L]

Patients also described feelings of sadness and depression (*n* = 6; 43%) or anguish and despair (*n* = 4; 29%), which accompanied their diagnosis or presented when they thought about both their current status and their future. Many patients had also witnessed parents and relatives suffer from the disease as they aged, giving rise to fear and anxiety about following a similar course themselves (*n* = 10; 71%).

Shock and despair [when I found out I had hATTR amyloidosis]. My whole world just—the doors shut. Because I knew what my dad had dealt with. And it was horrific. My dad was bed-bound for four years before he died at 49. And he was in a wheelchair for a couple years before that. And here I [was], at 60, extremely healthy except for this weird thing that nobody seem[ed] to be able to figure out. I mean, I had nothing else wrong with me. I had been very active in my life up until the symptoms of this caught up with me. So yes, it was pretty bleak. [#10, Female, Age 66, E54G]

### Impacts of hATTR amyloidosis on social functioning

Many patients (*n* = 11; 79%) described ways in which symptoms of hATTR amyloidosis affected their ability to engage in social activities. Impacts on social functioning included having far fewer opportunities to go out with their spouse or participate in events with their family and friends, often resulting in social isolation. Patients attributed these social impacts to a variety of sources, including difficulties walking, gastrointestinal dysfunction, fatigue, and pain.

Get-togethers with family and friends […] can be pretty difficult because they don’t see me as I used to be. I’m a little bit more crippled. And that can be difficult for my family to see, but also, just [not] being able to stay in the present, without letting the fatigue and brain fog take over. Because in longer periods of time being with people, the fatigue slowly sets in, and I just kind of start fading. And I’ve fallen asleep more than once on people, because it just slowly wears you down. […] My friends, we used to be very active. We like to kayak, we like to swim, we like go to down to the river and hang out. And some days, I just, I can’t. I used to be able to handle the heat. I can’t be outside in the summer anymore, so I can’t do that. But a lot of times, the pain is just too much. [#08, Female, Age 20, Y114H]

Difficulties walking and standing presented the biggest barrier to social participation for most patients. Many found that their physical instability meant that they had to give up social activities they enjoy such as going out to dinner, visiting with friends, going to a museum, or going shopping. Some shared fears of being unable to get to a bathroom quickly if necessary, or being unable to use restrooms that do not have a raised toilet seat, making most restrooms outside of their home inaccessible.

Issues with constipation and diarrhea, unexpected and just out of the blue. That’s tough to manage socially, to say the least. […] You have to prepare far in advance. As much as you can. […] I know where all the bathrooms are, let me tell you. I have had my share of emergencies where my husband is frantically trying to find a public restroom in a metro station, for instance. It’s no fun. […] [I would like] not having to worry about embarrassing myself in public. [#10, Female, Age 66, E54G]

Patients described how they had to give up physical activities they had enjoyed doing with others such as skiing, biking, walking, and ping pong. For some, it made participating in the activities that brought them joy and purpose impossible.

I [used to] coach basketball. And now I can’t. And there’s five [of my grandchildren] starting to play basketball, and I wanted to be able to show them things. Things like [that] really bother me. I mean, it’s not the end of the world, but I guess that would be the first thing, just to be able to have that balance back, and not worry about falling. [#06, Male, Age 73, V30M]

Some patients (*n* = 4; 29%) explained that their disease and its impacts were misunderstood by others. Friends and family would occasionally express frustration or confusion with patients when they were unavailable or unable to participate in activities that they were expected to. Two patients shared experiences of having difficulty communicating with others due to either physical deterioration of their voice or anxiety over cognitive deficits (*n* = 2; 14%), leaving them hesitant to engage in social activities. Others (*n* = 2; 14%) reported retreating socially, either because they felt distracted by the condition or simply desired to be alone.

### Impacts of hATTR amyloidosis on work and activities of daily living

Symptoms of PN and resulting impairments with gripping, holding, walking, or standing significantly interfered with patients’ ability to function in their daily lives. Many patients described having trouble performing ADLs such as dressing, bathing and using the bathroom independently (*n* = 9; 64%), household chores (*n* = 8; 57%), driving (*n* = 8; 57%), and eating (*n* = 3; 21%). Such impacts often resulted in patients’ loss of independence and a need for a caregiver to assist with ADLs.

When I lost my hand function, my activities of daily life became nearly impossible [to do]. So, I had to have a caregiver at home with me. Not because I needed medications or any medical stuff going on, or even because I couldn’t move around, because I can, but to do things like cook and clean, and anything requiring picking up stuff, and helping me in the shower, or helping me in the bathroom. You think about all of the things you use your hands for […] I mean there are some things that I figured out work-arounds [for], but most things, most activities of daily living I need help with. […] If I was able to [use my hands], I could do things that I want to do without having to have a caregiver. I could drive. I probably would be able to work again. And that would be very life changing. [#07, Male, Age 57, V30M]

Several of the patients who were employed when symptoms of hATTR amyloidosis began (*n* = 11; 79%) described how their work was impacted by their condition, including decreased productivity while at work (*n* = 7; 64%), frequent use of sick leave (*n* = 8; 73%), or needing to cease working altogether (*n* = 8; 73%). The loss of sensation and motor functioning in patients’ hands, limbs, or feet interfered with their ability to perform job-related duties and even contributed to hazardous situations while at work.

[I] took up a local job with a small company […] and it was that summer where I was walking on the golf course [and] started to limp. […] I was only with them for 13 months when I had to say that was it. I could not work. I went from being fully capable to having to hide because of this disability. […] Everything was fine to I just can’t work or walk [anymore] in a 13-month period. [#03, Male, Age 65, V30M]

Patients also related how gastrointestinal dysfunction or pain would contribute to presenteeism and absenteeism. One patient who was employed at the time of the interview described having to change jobs several times due to the disease.

I would have to call out pretty often. There were some days I was in so much pain, that just standing would make me cry. And so, we tried medications. They didn’t work. So, there were a lot of days I had to call out. And unfortunately, I ended up having to resign, due to my symptoms. And so, that was the first time. But it’s happened multiple times since then. [#08, Female, Age 20, Y114H]

Three patients (21%) had retired prior to the onset of hATTR amyloidosis symptoms. Though they did not experience work-related impacts due to the disease, each believed that they would not have been able to continue working had their symptoms emerged while still in the workforce.

## Discussion

Results from this study reveal that patients with hATTR amyloidosis have severe deficits across a broad range of physical, mental, and social functioning and well-being outcomes. Patients’ reports of disease burden in this study parallel findings from quantitative studies which have suggested that individuals with symptomatic hATTR amyloidosis experience significant impairment to quality of life [5, 7, 8]. This study expands on previous work by providing greater detail of patients’ experiences with the disease, detailing the nature of these impairments and the specific impacts on their lives.

Heterogeneity in clinical phenotype and the resemblance of symptoms to those of other common conditions can contribute to difficulty diagnosing this condition in patients [17]. The number of symptoms experienced by patients individually ranged from 4 to 15 total, and clusters of symptoms varied among patients. Patients interviewed for this study experienced worsening symptoms for nearly six years, on average, before receiving a diagnosis of hATTR amyloidosis.

This study had some limitations. Due to the challenges of recruiting patients with a rare disease, and the exploratory nature of this study, a convenience sampling approach was used, in that all eligible and interested patients who responded to recruitment flyers were included in the study. Thus, the study sample was heavily skewed with respect to education (college educated) and race (Caucasian/white). Improved health literacy and better earnings among highly educated patients could influence the ways they experience the disease and its impacts as well as the way they articulate those impacts. The lack of racial diversity in the study sample could limit the generalizability of the findings, as some genotypes more common to certain racial groups (e.g., the V122I variant among African Americans) may be associated with symptoms and impacts not fully represented in this study. Relatedly, sampling quotas for type of hATTR amyloidosis were not used and the study did not include any patients with *only* symptoms of CM; all patients had either symptoms of PN or symptoms of both PN and CM. The prevalence of patients with CM is estimated to be 5x the prevalence of patients with PN (50,000 patients worldwide, vs 10,000, respectively) [4], though those with CM *only* are thought to be largely underdiagnosed [1]. While a majority of patients in the study sample reported signs and symptoms of CM, co-occurrence with PN poses a challenge to identifying the specific impacts of CM on patients’ HRQoL. The absence of patients with only CM may mean that certain symptoms such as shortness of breath and swelling of the limbs, and the impacts of those symptoms, are also underrepresented in this study. Finally, similar to most qualitative research studies in rare diseases, the small sample size restricted the ability to investigate possible differences between subgroups of patients, such as between younger and older patients and those with early or late symptom onset. For example, while this study sample included one patient in their 20s and another in their late 40s, the rest of patients were over 50 years old. Further research could investigate whether the kind and magnitude of impacts from this disease differs between patients who experience symptoms earlier or later in their lives.

The methodological and analytic approach to this study has several strengths. Collaboration with an academic clinical center of excellence and a patient advocacy group allowed for confidence in the patient’s self-reported diagnosis. In addition, the patient population varied in terms of ambulatory disability stage and impacted organs/systems. This diversity supports the generalizability of these results to the greater population of patients with hATTR amyloidosis. Importantly, the use of individual, in-depth interviews allowed for a deep understanding of the burden of this disease. The symptoms reported by patients generally aligned with those found in literature, but the qualitative nature of this study allowed for connections to be drawn between symptoms and impacts on functioning and well-being. For example, patients provided rich descriptions of how numbness or loss of motor function resulted in having difficulties walking or gripping with their hands, which further led to deficits in their emotional-wellbeing and their social lives, as well as in their ability to carry out ADLs and perform at their jobs. The insights derived from these qualitative interviews extend the findings from quantitative studies by reporting, in the patients’ own words, the impacts from hATTR amyloidosis most salient to them and the variety of ways those impacts manifest in the contexts of their daily lives.

A 2018 consensus paper on the roadmap for advancing research on amyloidosis specifically noted that while several HRQoL measures have been used in clinical studies related to ATTR amyloidosis, each should undergo a comprehensive evaluation for relevance to patients with the disease, in addition to an examination of psychometric properties [18]. Identifying relevant and measurable concepts is an important first step to evaluating the content coverage of existing HRQoL patient-reported outcome (PRO) measures, and to identifying gaps that may require development of new, disease-specific PRO measures. Future efforts to develop a disease-specific PRO measure could build on the findings from this study in the creation of a conceptual framework of symptoms and impacts of hATTR amyloidosis [19]. In addition, this exploration of patients’ experiences with hATTR amyloidosis can help with identifying areas of unmet clinical need and ancillary care to patients, such as occupational therapy for workforce re-entry and other services to address deficits in patients’ ability to perform ADLs. This study, combined with recent publications from quantitative studies, is a contribution to these endeavors.

## Conclusions

The impacts of hATTR amyloidosis were profound for the patients interviewed for this study. They described a sense of loss as their condition progressed and impacted them physically, emotionally, and socially. Patients’ reports of symptoms and impacts of hATTR amyloidosis illustrate their complex and varied experiences of this disease. The progression of symptoms and increasing impacts of hATTR amyloidosis highlight the need for early and effective clinical intervention to preserve patients’ functioning and well-being.

## Data Availability

The data sets analyzed in the current study are available from the corresponding author on reasonable request.

## Abbreviations

ADLs: Activities of daily living
ASG: Amyloidosis Support Groups
BMC: Boston Medical Center
CM: Cardiomyopathy
hATTR: Hereditary transthyretin amyloidosis
HRQoL: Health-related quality of life
ICF: Informed consent form
NEIRB: New England Independent Review Board
PN: Polyneuropathy
PRO: Patient-reported outcome
SD: Standard deviation
TTR: Transthyretin
US: United States
